# Factors affecting hospital mortality rate in Iran: a panel data analysis

**DOI:** 10.1186/s13104-020-05410-w

**Published:** 2020-12-09

**Authors:** Mohsen Bayati, Mehrnoosh Emadi

**Affiliations:** Health Human Resources Research Center, School of Management & Information Sciences, Shiraz University of Medical Sciences, Almas Building, Alley 29, Qasrodasht Ave, Shiraz, Iran

**Keywords:** Hospital mortality, Death, Healthcare quality, Regression analysis, Panel data analysis

## Abstract

**Objective:**

Hospital deaths account for a large number of community deaths. Moreover, one of the main indicators of inpatient services quality is the hospital death. This study was performed to investigate the factors affecting hospital death rate in Iran using panel data analysis.

**Results:**

The net death rates in teaching and not-teaching hospitals were 6.24 and 5.58 per 1000 patients, respectively. Models' estimates showed, in teaching hospitals the number of surgeries (P < 0.05) and special beds (P < 0.01) had a significant positive relationship with death rate. In non-teaching hospitals, outpatient admissions (P < 0.01), number of surgeries (P < 0.05), number of special beds (P < 0.01), and length of stay (P < 0.01) had a positive and the number of inpatient admissions (P < 0.05) and active beds (P < 0.01) had a negative relationship with death rate.

Policy-making towards optimization of hospital service size and volume, standardization of length of stay, interventions to control nosocomial infections, and planning to control the complications of surgeries and anesthesia could effectively reduce hospital death rate.

## Introduction

The primary goal of health systems is to preserve, restore and promote the health of individuals in societies. One of the main indicators of health status is mortality [1]. From an epidemiological point of view, death is one of the most important measures of health outcomes in any community [2].

Accurate and timely statistics on mortality indicators appear essential for policy making in the health sector [3]. In other words, obtaining precise and well-timed information and statistics on mortality is one of the most fundamental principles for planning and setting health priorities in any health system [4].

Today, hospital deaths account for a large number of community deaths [5]. More than 54% of the deaths worldwide occur in hospitals; Japan and China accounted for the highest (78%) and lowest (20%) hospital deaths of overall deaths in 2013 [6].

Moreover, one of the indicators of services quality is the hospital death rate [7] and the data of which are commonly used to assess the quality of inpatient services [8]. Valid statistics and information on the trend and causes of hospital deaths are an important basis for determining the effectiveness of health care interventions designed to promote health [9]. In addition, end-of-life care and hospital deaths are among the major causes of increased health care costs [10].

According to few studies conducted, hospital mortality can be associated with the structural and organizational characteristics of hospitals such as type of hospital, number of surgeries, inpatient and outpatient admissions, type of hospital specialty, length of patient stay, and other factors [11].

This study was carried out to investigate the factors affecting hospital death. The results can be used in future planning by health policy makers to reduce hospital mortality rates.

## Main text

### Methods

In the current research, the entire research population, i.e. the hospitals affiliated to Shiraz University of Medical Sciences, was studied. The required data were collected from 32 hospitals over a 24-month period, from March 2015 to February 2017. So all observation was 768. For analysis, hospitals were categorized into teaching and non-teaching hospitals. Thus 21 teaching hospitals (504 observations) and 11 non-teaching ones (264 observations) were included.

A researcher-made checklist was used to collect the data, and the required information was extracted from the hospitals’ statistical systems. We have no missing data.

The response variable in this study was hospital deaths. We used the hospital net death rate which refers to the total number of deaths occurring after 24 h of admission compared to the total deaths and discharges after 24 h of admission per one thousand.

The variables affecting hospital mortality were selected based on previous studies and data availability.

$$ {\text{NHDR }} = {\text{ F }}\left( {{\text{OA}},{\text{ IA}},{\text{ SO}},{\text{ LOS}},{\text{ SB}},{\text{ AB}}} \right) $$

As shown in the above function, net hospital death rate (NHDR) is affected by outpatient admission (OA), inpatient admission (IA), surgical operations (SO), length of stay (LOS), special bed (SB) and active bed (AB).

With respect to our data, following panel data model was developed:

$$ {\text{NHDR}}_{{{\text{it}}}} = \beta_{{{\text{i}}0}} + \beta_{{1}} {\text{OA}}_{{{\text{it}}}} + \, \beta_{{2}} {\text{IA}}_{{{\text{it}}}} + \, \beta_{{3}} {\text{SO}}_{{{\text{it}}}} + \, \beta_{{4}} {\text{LOS}}_{{{\text{it}}}} + \, \beta_{{5}} {\text{SB}}_{{{\text{it}}}} + \, \beta_{{6}} {\text{AB}}_{{{\text{it}}}} + {\text{U}}_{{{\text{it}}}} $$

where, i, t and u show hospital, month and regression error term, respectively. B0-B6 also indicate the models coefficients.

To choose estimation method, paned data model diagnostic measures including Chow, Breusch-Pagan and Hausman tests were performed.

Analysis was conducted by STATA 14.

### Results

According to the findings of this study, the death rates per 1000 patients in teaching hospitals (6.24) were higher than in non-teaching ones (5.58). The descriptive statistics of other studied variables are presented in Table 1 on a monthly basis by teaching and non-teaching hospitals.

**Table 1:** Descriptive finding of net hospital death rate and other hospitals' characteristics

Variables	Educational hospitals		Non-educational hospitals	
	Mean	S.D	Mean	S.D
Net hospital death rate (per 1000 population)	6.24	7.75	5.58	6.27
Outpatient admission	271.47	413.40	284.89	461.02
Surgical operations	390.70	366.65	284.40	283.54
Special bed	17.05	13.58	14.51	9.85
Length of stay	5.48	9.37	8.26	12.23
Inpatient admission	877.98	646.94	826.28	694.44
Active bed	135.40	102.65	150.69	101.61

The results of the diagnostic tests for panel data model are shown in Table 2. As summary, there are three diagnostic tests for selection of estimation method. The Chow test specifies the best model between the pooled OLS (null hypothesis) and the fixed effect (alternative hypothesis). The Breusch and Pagan Lagrangian multiplier determines the most suitable method among pooled OLS (null hypothesis) and random effects model (alternative hypothesis). The Hausman test is used to select the appropriate model between the random effects (null hypothesis) model and the fixed effects model (alternative hypothesis) [12]. In teaching hospitals, as the results indicated the null hypothesis (pooled effects) of the Chow test and of the Breusch–Pagan test were rejected. Therefore, the Hausman test is performed for selection between random and fixed effect models. According to it null hypothesis (random effects) not rejected and the random effects method was chosen to estimate the model.

**Table 2:** Diagnostic tests for choosing estimation method

Diagnostic tests	Educational hospitals	Non-educational hospitals
Fixed effects test	17.12 (0.0000)	4.41 (0.0005)
Breusch-pagan	419.38 (0.0000)	3.76 (0.0523)
Hausman test	11.49 (0.0743)	–

In the non-teaching hospitals, the fixed effects method was used for estimating the model based on the results of the Chow and Breusch–Pagan tests.

The model estimation results are shown in Table 3. In teaching hospitals, the number of surgeries and number of special beds showed a significant positive relationship with death rate. Other variables (outpatient admissions, length of stay, inpatient admissions, and number of active beds) were not significantly related to death rate.

**Table 3:** Estimates of factors affecting hospital death rate in educational and non-educational hospitals

Explanatory variables	Educational hospitals		Non-Educational hospitals	
	Coefficient	P-Value	Coefficient	P-Value
Outpatient admission	0.000876	0.2531	0.003913	0.0008
Surgical operations	0.006349	0.0108	0.007162	0.0208
Special Bed	0.452609	0.0000	0.517404	0.0000
Length of stay	0.450332	0.1680	1.227151	0.0002
Inpatient admission	− 0.000264	0.7898	− 0.003731	0.0347
Active bed	− 0.015911	0.1295	− 0.033686	0.0078
Overall significance and goodness of fit	F-statistic: 97.87 P-Value = 0.000		F-statistic: 29.23 P-Value = 0.000	
	Adjusted R-squared = 0.68		Adjusted R-squared = 0.56	

In non-teaching hospitals, outpatient admissions, number of surgeries, number of special beds, and length of stay had a positive significant relationship with death rate. There was also a negative relationship between mortality and the number of inpatient admissions and active beds.

Given the value of the F statistic, the overall significance of models was confirmed. Besides, the results of adjusted coefficient of determination showed that in teaching and non-teaching hospitals, about 68% and 56% of the changes in hospital death rate could be explained by the regression line fitted to the variables under study.

### Discussion

Given the remarkable proportion of hospital deaths among all deaths, the aim of this study was to determine the factors affecting hospital death rate in Iran.

In this study, the mean of death rate in teaching hospitals was higher than in non-teaching ones. In this regard, various studies reported different results.

Golikov showed that in the US, mortality rate in teaching hospitals (4.64%) were higher than in non-teaching ones (3.68%) [13]. In a study by Patel in the US, mortality rate in non-teaching hospitals were lower than in teaching ones [14] and the results of the study by Cram in California showed that the mortality rate in hospitalized patients in teaching hospitals was higher than in non-teaching ones on weekends [15]. On the other hand, Allison carried out a study in 4361 hospitals in the US and found that acute MI patients in teaching hospitals had lower mortality rate than in non-teaching ones, and the more powerful a hospital was in terms of knowledge and education, the lower the death rate would be [16]. The results of the study by Burke in the US showed that under normal conditions, the death rate in teaching hospitals was lower than in non-teaching ones [17], and Hartz indicated that in the US, adjusted mortality rates in private teaching hospitals were lower (108 per 1000) than in non-teaching ones (116 per 1000) [18].

In the present study, the result (higher death rate in teaching hospitals) could be justified because teaching hospitals were usually the main and referral centers for specific/super specialized medical services and complex patients compared to other hospitals. In this regards, some studies stated that sicker and more complex patients are usually admitted in teaching hospitals [19].

The differences might also be due to a variety of factors, such as different hospital death rate definitions (some studies examined hospital mortality rate for particular diseases), differences in the concept of teaching/non-teaching hospitals, and other factors related to the health system and socioeconomic factors as well.

According to the analytical findings, factors such as outpatient admission, number of surgeries, number of special beds, and length of stay were among the factors affecting hospital death rate.

Before discussion about findings of regression estimates, an important issue should be debated. Expect from special bed and length of stay some coefficients are significant but they are very low. It shows that they have very low explanation of hospital death rate. Although, it tried to include the suitable explanatory factors according to the literature but limitation to access to valid data restricted the model to current variables. In this regard, factors such as service quality, specialty of hospital, severity of illness, type of illness, age of patients and level of hospital care could be considered as determinants of hospital death in future studies. Further research especially at patient level with higher sample size can provide more opportunity to estimate more comprehensive model for hospital death.

In teaching and non-teaching hospitals, the number of surgeries had a positive and significant relationship with the hospital death rate. As surgeries were usually performed on people with acute conditions such as accidents and injuries and had also significant complications, the individuals were more likely to die than by other hospital services. The deaths occurred for various reasons including a disease or injury for which a surgery was performed, deaths due to anesthesia complications or mistakes, deaths due to surgery complications or mistakes, or diagnostic procedures. In the study by Akhlaghi [20] in Tehran, 9% of all deaths following surgeries were due to surgical and anesthetic factors. In a study in Mackay, it is stated that the majority of hospital deaths (55%) followed emergency surgeries [21].

There was a significant and positive relationship between the number of special beds and the hospital death rate. As critically ill patients were usually admitted to these wards, the risk of dying in hospitals with more special beds was higher. Similar studies reported significant mortality rates in intensive care units. In a study in Mexico (1998–2002), the crude mortality rate in the intensive care units was 27% [22]. The mean Intensive Care Unit (ICU) mortality rate reported in the United States ranged from 8 to 19% or about 500,000 deaths per year [23]. Alam conducted a study in Bangladesh and showed that the number of ICU beds accounted for 1.8% of the total hospital beds; however the death rate in the ICU was 3.58% of hospital deaths [24]. According to Siddiqui, there were 1134 deaths in three ICU wards in Singapore from July 2010 to March 2015, accounting for 7% of all hospital deaths. The mean mortality rates in the Medical Intensive Care Unit (ICU), Surgical Intensive Care Unit (SICU), and Coronary Care Unit (CCU) were 7%, 8.5%, and 8.2%, respectively [25].

In non-teaching hospitals, the length of stay showed a positive and significant relationship with hospital death rate. Prolonged hospital stay not only exposed the patient to a variety of nosocomial infections and psychological trauma, but also posed a threat to his/her mental and physical health and caused physical weakness and ultimately death. The Centers for Disease Control and Prevention (CDC) estimated that in the US, about 99,000 deaths each year were due to healthcare-related infections in hospitals [26]. Morgan indicated that 55 (31%) out of 179 unexpected hospital deaths in a health center in Baltimore within 2004–2008 were due to healthcare-related infections, which accounted for almost one-third of all unexpected deaths in hospitals [27].

The findings of this study were consistent with those of the Al-Haider in Saudi Arabia [28] and Lingsma in six developed countries [29], showing that increased length of stay would increase mortality rates. In a 12-year study in Japan, Nakamura found that patients who stayed in intensive care units for more than two weeks had significantly higher mortality rates [30].

In non-teaching hospitals, the number of hospital admissions and active beds were negatively correlated with death rate. Various studies reported different results regarding the impact of hospitalization service volume on hospital death rate. For example, a study in the US showed that increased admissions led to a decreased number of deaths [31]. Yoshioka showed that there was a significant relationship between increased service volume and lower mortality rates in Japanese hospitals [32]. Fareed showed that in the US, patients in larger hospitals were less likely to die than those admitted to smaller hospitals. Besides, the probability of death in large hospitals was 11% lower than in small ones [33]. But a study conducted in Saudi Arabia showed that the hospital mortality rate increased as hospital size and the number of hospital beds increased [28]. Also, Kelly showed that in the US, hospital mortality rates increased with the hospital size and hospitalization rate [34].

Given that the present study mainly examined the organizational factors affecting hospital deaths, it can help managers and policy makers to make evidence-based decisions in order to control hospital deaths. Another strength of this study is the use of hospital data over time (panel data), which makes the study findings more reliable than those of cross-sectional or time series studies.

### Conclusion

The mortality rate in teaching hospitals was higher than in non-teaching ones. According to the results, policy-making towards the development of primary care such as proper implementation of the family physician program and referral system, optimization of hospital service size and volume, standardization of length of stay, interventions to control nosocomial infections, and planning to control the complications of surgeries and anesthesia could effectively reduce hospital death rate.

## Limitations

Despite the strengths mentioned, the study had some limitations, one of which was the uncertainty about the quality of hospital data. We used the hospital statistics system information that might not be accurate and complete enough. The other limitation of the study was that many variables could affect hospital death which could not be controlled for their effects due to the lack of access to information, incomplete data, and the problems with model estimation. Another limitation was that some estimated coefficients were significant but they were very low, i.e. they have low explanation of hospital death rate. So they should be interpreted with caution.

## Data Availability

The data used in this study are not publicly available because the participants were promised that the raw data would remain confidential. However they are available from the corresponding author on reasonable request.

## Abbreviations

AB: Active bed
CCU: Coronary care unit
CDC: Centers for disease control and prevention
IA: Inpatient admission
ICU: Intensive care unit
LOS: Length of stay
MICU: Medical intensive care unit
NHDR: Net hospital death rate
OA: Outpatient admission
SB: Special bed
SICU: Surgical intensive care unit
SO: Surgical operations
